# Demographic, Clinical, and Biochemical Characteristics of Patients Admitted With Diabetic Ketoacidosis (DKA): A Retrospective Descriptive Study

**DOI:** 10.7759/cureus.88882

**Published:** 2025-07-28

**Authors:** Abdulrahman Tabrah, Afnan Al-Bakri

**Affiliations:** 1 Internal Medicine, Al-Karkh Baghdad Health Directorate, Baghdad, IRQ

**Keywords:** clinical presentation of dka, dka: diabetic ketoacidosis, dka manifestation, precipitating factors of dka, urinary tract infection dka

## Abstract

Background: Diabetic ketoacidosis (DKA) and hyperglycemic hyperosmolar state (HHS) are serious acute complications of diabetes associated with substantial morbidity and mortality worldwide, particularly in low- and middle-income countries. DKA disproportionately affects younger individuals with type 1 diabetes and remains a major cause of diabetes-related hospital admissions and deaths in resource-limited settings like Iraq.

Objective: This study aimed to assess the clinical presentation, precipitating factors, and in-hospital outcomes of patients admitted with DKA to hospitals under the Al-Karkh Health Directorate in Baghdad, Iraq.

Methods: We conducted a retrospective review of 200 patients (aged ≥16 years) admitted with DKA between January and June 2025. Data collected included demographics, clinical features, laboratory findings, DKA triggers, management, and outcomes. Descriptive statistics were used to report findings in frequency and percentage format.

Results: The majority of patients were young adults (87.5% aged ≤30 years) with a slight male predominance (55%). Type 1 diabetes was the most common diagnosis (61.5%), while 31.5% were newly diagnosed at presentation. The leading triggers of DKA were insulin non-compliance (50.5%) and infection (35%). Most patients presented with gastrointestinal symptoms (81.5%), while 16% exhibited confusion and 2.5% were in septic shock. Electrolyte disturbances were common, with 43.5% of patients exhibiting abnormal potassium levels. Intensive care unit (ICU) admission was required in 10.5% of cases, and the in-hospital mortality rate was 3.5%.

Conclusion: DKA remains a major health burden among young adults with type 1 diabetes in Baghdad. The most common precipitating factors were missed insulin doses and infections. Strengthening patient education, improving access to insulin, and promoting early symptom recognition are crucial strategies to reduce DKA-related complications and mortality.

## Introduction

Diabetes is a lifelong condition that millions of individuals manage daily, yet when it becomes unstable, the resulting complications can be life-threatening. Acute hyperglycemic emergencies such as diabetic ketoacidosis (DKA) and hyperglycemic hyperosmolar state (HHS) serve as critical reminders, not only to patients and their families but also to healthcare systems, of the serious burden this disease can impose.

DKA and HHS are the most severe manifestations of hyperglycemic crisis, driven by insulin deficiency and compounded by elevations in counterregulatory hormones such as glucagon, cortisol, catecholamines, and growth hormone. Although these conditions share overlapping clinical features, such as dehydration, altered consciousness, and hyperglycemia, DKA is typically defined by a triad of hyperglycemia, ketonemia, and metabolic acidosis and is more prevalent in individuals with type 1 diabetes. HHS, in contrast, occurs more often in older adults with type 2 diabetes and presents with extreme hyperglycemia and hyperosmolality without significant ketoacidosis [1,2].

The pathophysiology of DKA involves a catabolic state induced by absolute insulin deficiency, which leads to uncontrolled lipolysis, accumulation of ketone bodies, acidosis, osmotic diuresis, and profound electrolyte and volume depletion [3-5]. In many cases, these crises are precipitated by preventable factors such as missed insulin doses, intercurrent infections, or a lack of understanding about insulin adjustment during illness, especially in socioeconomically disadvantaged regions.

Effective management of DKA requires prompt recognition, fluid resuscitation, insulin administration, correction of electrolyte disturbances, and identification of underlying triggers. In pediatric cases, heightened vigilance is required to prevent complications such as cerebral edema, making timely intervention critical [1,6,7].

While progress in DKA management has improved survival rates globally, prevention remains a persistent challenge, particularly in resource-limited settings. In countries like Iraq, ongoing systemic barriers, ranging from economic instability and limited healthcare infrastructure to regional conflict and disrupted insulin supply chains, compound the difficulties of managing chronic conditions like diabetes [5,6]. Public awareness campaigns, structured diabetes education, and reliable access to essential medications remain inconsistent, leaving many patients vulnerable to life-threatening complications.

This study aims to provide much-needed local epidemiological data on DKA in Baghdad, Iraq. By evaluating patient demographics, clinical presentation, precipitating factors, and hospital outcomes, we seek to better understand the burden of DKA in this specific context. Our findings are intended to inform prevention strategies, enhance early recognition and management protocols, and ultimately improve outcomes for diabetic patients in Baghdad and similar resource-constrained settings.

## Materials and methods

Study design and setting

This was a retrospective observational study conducted across hospitals affiliated with the Al-Karkh Health Directorate in Baghdad, Iraq. Medical records of patients admitted with diabetic ketoacidosis (DKA) were reviewed over six months, from January 1 to June 30, 2025. The study aimed to assess clinical characteristics, biochemical abnormalities, precipitating factors, and in-hospital outcomes to provide insight into the local burden and management patterns of DKA.

Inclusion and exclusion criteria

To ensure a focused and meaningful analysis, we carefully selected patients whose records met specific clinical and laboratory criteria. We included individuals aged 16 years or older who were diagnosed with diabetic ketoacidosis (DKA) upon hospital admission. These diagnoses were based on the clinical judgment of treating physicians, supported by laboratory evidence, specifically, a serum bicarbonate level of less than 18 mmol/L, indicating metabolic acidosis.

We also required that each patient had a complete set of essential biochemical results, including serum potassium, creatinine, and white blood cell (WBC) count, as well as a documented hospital outcome, whether they survived or, unfortunately, passed away. These parameters were critical for building a comprehensive picture of each case.

On the other hand, we excluded patients from the study if they were diagnosed with a different form of hyperglycemic crisis, such as hyperglycemic hyperosmolar state (HHS), were under the age of 16, or if their medical records lacked key information, such as vital lab results or outcome data. This helped maintain the integrity and consistency of our analysis.

Data collection

Records were carefully reviewed by trained staff who manually extracted key pieces of information that would help paint a comprehensive picture of each patient’s journey through diabetic ketoacidosis (DKA).

We began by gathering demographic details, such as the patient’s age, sex, body mass index (BMI), smoking status, and whether they came from an urban or rural setting. From there, we explored their diabetes history, including the type of diabetes they were living with (type 1, type 2, or newly diagnosed) and whether they had previously experienced any episodes of DKA.

We also looked closely at how each patient presented upon admission. This included identifying possible triggers like infections or missed insulin doses, along with symptoms, level of consciousness as measured by the Glasgow Coma Scale (GCS), and their blood pressure status.

Laboratory findings played a crucial role in confirming the diagnosis and understanding the severity of each case. We collected data on random blood glucose levels, serum pH, ketones in urine, bicarbonate (HCO₃), serum potassium (K⁺), creatinine levels, and white blood cell (WBC) count.

Finally, we recorded important outcomes, whether the patient required care in the intensive care unit (ICU) and whether they survived or died during hospitalization.

Statistical analysis

Once the data were collected, we used descriptive statistics to summarize the findings and bring out the key patterns. Categorical variables, such as the type of diabetes, presenting symptoms, and whether ICU care was required, were reported in terms of frequency (N) and percentage (%). All data were organized and analyzed using Microsoft Excel (Redmond, USA). Since this was a purely descriptive study focused on identifying trends and patterns rather than testing hypotheses, we did not apply inferential statistical tests.

## Results

A total of 200 patients diagnosed with diabetic ketoacidosis (DKA) were included in this study. All patients met the inclusion criteria and had complete medical records available for analysis.

Demographic characteristics 

The majority of patients were young adults: 60 (30.0%) were aged 16-20 years, 115 (57.5%) were aged 21-30 years, and only 25 (12.5%) were older than 30. Of the total sample, 110 (55.0%) were male and 90 (45.0%) were female. Body mass index (BMI) was normal in 140 (70.0%), while 22 (11.0%) were overweight, 13 (6.5%) were obese, and 24 (12.0%) were underweight. A majority of patients, 155 (77.5%), came from rural areas, while 45 (22.5%) were from urban settings. Smoking was prevalent, with 130 (65.0%) identified as current smokers and 70 (35.0%) as non-smokers (Table 1).

**Table 1:** Demographic variables BMI: Body mass index

Variable	Categories	Number of Patients (n)	Percentage (%)
Age (years)	16–20	60	30.0%
NA	21–30	115	57.5%
NA	>30	25	12.5%
Sex	Male	110	55.0%
NA	Female	90	45.0%
BMI (kg/m²)	Normal	140	70.0%
NA	Overweight	22	11.0%
NA	Obese	13	6.5%
NA	Underweight	24	12.0%
Residence	Rural	155	77.5%
NA	Urban	45	22.5%
Smoking Status	Current Smoker	130	65.0%
NA	Non-smoker	70	35.0%

Diabetes history 

Regarding diabetes classification, 123 (61.5%) had established type 1 diabetes, 63 (31.5%) were newly diagnosed at the time of presentation, and 14 (7.0%) had type 2 diabetes. A total of 97 (48.5%) experienced previous episodes of DKA, while 103 (51.5%) were presenting for the first time (Table 2).

**Table 2:** Diabetes history DKA: Diabetic ketoacidosis

Variable	Categories	Number of Patients (n=200)	Percentage (%)
Type of Diabetes	Type 1	123	61.5
NA	Newly Diagnosed	63	31.5
NA	Type 2	14	7.0
Previous DKA Episodes	Yes	97	48.5
NA	No	103	51.5

Clinical presentation 

The most common precipitating factor for DKA was insulin non-compliance, reported in 101 (50.5%) of patients. This was followed by infections in 70 (35.0%), dietary nonadherence in 22 (11.0%), and myocardial infarction in seven (3.5%). Most patients (163; 81.5%) presented with classical symptoms such as nausea, vomiting, or abdominal pain. However, 32 (16.0%) exhibited confusion, and five (2.5%) presented in septic shock. Consciousness level at admission, assessed using the Glasgow Coma Scale (GCS), was normal (GCS = 15) in 163 (81.5%), mildly reduced (GCS = 14) in 29 (14.5%), and significantly impaired in eight patients (4.0%), of whom three (1.5%) had GCS = 10 and five (2.5%) had GCS < 8. At presentation, 137 (68.5%) were normotensive, while 63 (31.5%) were hypotensive (Table 3).

**Table 3:** Clinical presentation GCS: Glasgow Coma Scale, DKA: Diabetic ketoacidosis

Variable	Categories	Number of Patients (n=200)	Percentage (%)
Precipitating Factor	Infection	70	35.0
NA	Myocardial Infarction (MI)	7	3.5
NA	Insulin Non-compliance	101	50.5
NA	Poor Diet	22	11.0
Presenting Symptoms	Typical DKA (N/V, abdominal pain)	163	81.5
NA	Confusion	32	16.0
NA	Septic Shock	5	2.5
GCS on Admission	15	163	81.5
NA	14	29	14.5
NA	10	3	1.5
NA	<8	5	2.5
Blood Pressure	Normotensive	137	68.5
NA	Hypotensive	63	31.5

Biochemical findings 

Random blood glucose levels varied, with 107 (53.5%) having values between 300-400 mg/dL. Normoglycemic DKA was noted in 10 (5.0%), while 25 (12.5%) had glucose levels between 400-500 mg/dL, 28 (14.0%) between 500-700 mg/dL, 17 (8.5%) between 700-800 mg/dL, and 13 (6.5%) exceeding 800 mg/dL. Blood gas analysis revealed metabolic acidosis (pH < 7.35) in 177 (88.5%) patients, while 23 (11.5%) had near-normal pH (7.35-7.45) accompanied by low carbon dioxide levels.

All 200 (100%) patients had low bicarbonate (HCO₃⁻) levels and more than +3 ketones in urine, consistent with acidosis. Serum potassium levels were normal (3.5-5.5 mmol/L) in 113 (56.5%), elevated (>5.5 mmol/L) in 55 (27.5%), and low (<3.5 mmol/L) in 32 (16.0%). Serum creatinine was within normal range in 190 (95.0%), but elevated in 10 (5.0%), suggesting potential renal impairment. White blood cell (WBC) counts were raised in 74 (37.0%), which may reflect infection or a stress response, and were normal in the remaining 126 (63.0%) (Table 4).

**Table 4:** Biochemical findings

Parameter	Category / Range	Reference Range	Number of Patients (n)	Percentage (%)
Random Blood Glucose	Normoglycemic DKA	70–140 mg/dL	10	5.0%
NA	300–400 mg/dL	>140 mg/dL	107	53.5%
NA	400–500 mg/dL	>140 mg/dL	25	12.5%
NA	500–700 mg/dL	>140 mg/dL	28	14.0%
NA	700–800 mg/dL	>140 mg/dL	17	8.5%
NA	>800 mg/dL	>140 mg/dL	13	6.5%
Serum pH	<7.35 (Metabolic Acidosis)	7.35–7.45	177	88.5%
NA	7.35–7.45 with low pCO₂ (compensated)	7.35–7.45	23	11.5%
Bicarbonate (HCO₃⁻)	Low in all patients	22–28 mmol/L	200	100.0%
Serum Potassium (K⁺)	Normal	3.5–5.5 mmol/L	113	56.5%
NA	Elevated	>5.5 mmol/L	55	27.5%
NA	Low	<3.5 mmol/L	32	16.0%
Serum Creatinine	Normal	0.6–1.2 mg/dL	190	95.0%
NA	Elevated	>1.2 mg/dL	10	5.0%
White Blood Cell Count	Normal	4,000–11,000 /mm³	126	63.0%
NA	Elevated	>11,000 /mm³	74	37.0%
Urinary Ketones	+3 or more	Negative to Trace	200	100.0%

Management and outcomes 

Only 21 (10.5%) of the patients required admission to the intensive care unit (ICU), indicating that most cases were managed on standard medical wards. In-hospital mortality occurred in seven (3.5%), while 193 (96.5%) of the patients survived and were discharged after appropriate treatment (Table 5).

**Table 5:** Management and outcomes ICU: Intensive care unit

Variable	Categories	Number of Patients (n=200)	Percentage (%)
ICU Admission	Yes	21	10.5
NA	No	179	89.5
In-hospital Outcome	Survived	193	96.5
NA	Died	7	3.5

## Discussion

Our study highlights that diabetic ketoacidosis (DKA) predominantly affects young adults living with type 1 diabetes, with 123 (61.5%) of the 200 patients affected, a pattern that mirrors findings from other research [1]. Interestingly, we observed a slightly higher number of males (110, 55%) affected and a considerable proportion of smokers (130, 65%) among our patients. These factors may reflect cultural or social influences that affect how individuals manage their diabetes, similar to patterns seen in other studies involving children and adolescents [2].

What stood out most in our findings was that just over half of the patients, 101 (50.5%), developed DKA due to missed insulin doses. This finding aligns with global data, which consistently highlights poor adherence to insulin therapy as the leading cause of DKA episodes [3]. Infections followed as the second most common trigger, affecting 70 (35%) patients, reinforcing the well-established link between intercurrent illness and metabolic decompensation in individuals with diabetes [4].

In our setting, several factors likely contributed to insulin non-compliance. While detailed psychosocial data were limited in the charts, documentation frequently cited financial barriers, irregular access to insulin, and lack of structured diabetes education as contributing factors. Some patients also expressed confusion about how to adjust insulin during illness, and a few charts referenced fear of hypoglycemia. These challenges highlight the urgent need for multifaceted interventions, including improving insulin availability, expanding patient education, and establishing reliable follow-up systems, to reduce the risk of preventable DKA admissions in this population.

Clinically, most patients presented with the classic symptoms of DKA: excessive thirst, frequent urination, and abdominal pain, which were present in 163 (81.5%) of patients and are well recognized in standard clinical guidelines [1,4]. However, some patients arrived in a much more serious condition, exhibiting confusion (32, 16%) or even septic shock (5, 2.5%). This range of severity underscores the critical importance of recognizing early warning signs and responding swiftly to prevent life-threatening complications [1].

Our laboratory findings painted a picture of significant acid imbalance in the blood, with 177 (88.5%) patients having a serum pH below 7.35, along with common electrolyte disturbances, especially involving potassium, where 55 (27.5%) had elevated potassium levels. These results are consistent with what is known about how DKA affects the body and highlight the need for careful monitoring throughout treatment [3,7,8]. Despite many patients showing considerable biochemical abnormalities, only 21 (10.5%) required intensive care. This may reflect local healthcare capacities or specific ICU admission criteria. Encouragingly, the mortality rate was low at seven (3.5%), which matches international reports and suggests that adherence to proper treatment protocols can greatly reduce fatal outcomes [1,7].

One of the key strengths of our study is the comprehensive dataset gathered from a busy hospital setting, offering valuable real-world clinical insights. Nevertheless, the retrospective design, focus on a single healthcare region, and absence of long-term follow-up data limit the generalizability of our findings. These are common challenges shared by similar studies and point to the need for future research with broader and more extended patient monitoring. Additionally, selection bias is a potential concern, as the study only included hospitalized patients with documented DKA, potentially overlooking milder cases managed in outpatient settings or undiagnosed presentations. These are common challenges shared by similar studies and point to the need for future research with broader and more extended patient monitoring [5,6].

## Conclusions

Diabetic ketoacidosis (DKA) remains a serious and potentially life-threatening complication of diabetes, particularly among young people with type 1 diabetes. In our study, the leading trigger was insulin non-compliance, accounting for 50.5% of cases, followed by infections at 35%. These findings align with global data and underscore the urgent need for targeted, evidence-based interventions. Given our finding that insulin non-compliance was the primary precipitating factor, education plays a truly critical role in DKA prevention. Structured programs aimed at improving patients’ understanding of insulin therapy, recognizing early warning signs, and managing sick days could significantly reduce the frequency and severity of DKA episodes. These efforts should also involve caregivers, especially for adolescents and young adults, where adherence may be more variable.

Equally important is ensuring consistent access to affordable insulin. In regions like Baghdad, where systemic challenges, including supply interruptions, economic hardship, and limited diabetes support infrastructure, exist, public health policies must prioritize equitable insulin distribution and access. Our findings also highlight the importance of robust clinical protocols to identify and manage common DKA triggers such as infection. Multidisciplinary coordination among endocrinologists, diabetes educators, emergency physicians, and primary care teams is essential for delivering timely care and preventing recurrence. Meeting the complex challenge of DKA in resource-limited settings requires a comprehensive and collaborative approach that combines patient education, improved healthcare access, and integrated care delivery. Future research should focus on evaluating the effectiveness of targeted educational interventions and novel insulin delivery systems in reducing non-compliance-related DKA episodes. A prospective or interventional study in our setting could provide valuable insights into how to best support high-risk populations and prevent avoidable hospitalizations. By translating our findings into action, we can reduce the burden of DKA on patients, families, and the healthcare system and ultimately improve outcomes for people living with diabetes.

## References

[1] Kitabchi AE, Umpierrez GE, Miles JM, Fisher JN (2009). Hyperglycemic crises in adult patients with diabetes. Diabetes Care.

[2] DiMeglio LA, Acerini CL, Codner E (2018). ISPAD Clinical Practice Consensus Guidelines 2018: Glycemic control targets and glucose monitoring for children, adolescents, and young adults with diabetes. Pediatr Diabetes.

[3] Nyenwe EA, Kitabchi AE (2016). The evolution of diabetic ketoacidosis: An update of its etiology, pathogenesis and management. Metabolism.

[4] Umpierrez GE, Murphy MB, Kitabchi AE (2002). Diabetic ketoacidosis and hyperglycemic hyperosmolar syndrome. Diabetes Spectrum.

[5] Ahuja W, Kumar N, Kumar S, Rizwan A (2019). Precipitating risk factors, clinical presentation, and outcome of diabetic ketoacidosis in patients with type 1 diabetes. Cureus.

[6] Bismuth E, Laffel L (2007). Can we prevent diabetic ketoacidosis in children?. Pediatr Diabetes.

[7] Fayfman M, Pasquel FJ, Umpierrez GE (2017). Management of hyperglycemic crises: Diabetic ketoacidosis and hyperglycemic hyperosmolar state. Med Clin North Am.

[8] Eledrisi MS, Alshanti MS, Shah MF (2006). Overview of the diagnosis and management of diabetic ketoacidosis. Am J Med Sci.

